# The King's Hospital Fund

**Published:** 1902-11-29

**Authors:** 


### The Hospital.

A Journal of

The Medical Sciences and Hospital Administration.

Vol. XXXIII.—No. 844.] Edited by Sir Henry Burdett, K.C.B., and Solomon C. Smith, M.D., M.R.C.P. [November 29, 1902.

### THE KING'S HOSPITAL FUND.

The account we publish in another column of the meeting of the Council of the King Edward's Hospital Fund on Monday last will be memorable in the history of voluntary hospitals. It was very brave of his Majesty the King, when Prince of Wales, to place himself at the head of the movement to raise by collective efforts from £100,000 to £150,000 of additional income for the London hospitals. The proposal was regarded with anything but favour by a large number of people representing various classes of opinion, the fear being that success was altogether out of the question. It was urged that the monarchy ought only to identify itself with established institutions the success of which was assured. With a courage and prescience for which King Edward will always be gratefully remembered he threw himself into the movement on behalf of the hospitals in commemoration of Queen Victoria's Diamond Jubilee. It must therefore be gratifying to his Majesty that the Prince of Wales has taken so active an interest in the King's Fund and that in the first year of his presidency £100,000 has been distributed amongst the hospitals. We believe this success to be due in no small degree to the recognition of the privilege of personal service in the cause of the sick, which the King and Queen, and the Prince and Princess of Wales especially, have believed in and acted upon as a principle of daily life. We tender to King Edward and to H.R.H. the President of the Fund the sincerest congratulations and warmest thanks of all who are interested in our hospitals upon the gratifying success attained by King Edward's Hospital Fund, mainly through their example and interest.

The financial success of the King's Fund is now assured. The Prince states that an additional £10,000 a year will ensure the distribution next year of a second £100,000 amongst the hospitals. The League of Mercy has been extending its work and influence with gratifying success, and we have reason to believe that within the next five years the income of the King's Fund will certainly be not less than £150,000 a year. The Committee of Distribution have been authorised by the Council, on their own initiative, to labour to secure the amalgamation of some of the smaller hospitals. Such a movement must diminish the cost of management make the distribution of hospital beds more in accordance with the requirements of the population of London, secure for the institutions amalgamated a larger measure of public support and confidence, and generally be fruitful in results for good. That the Council should determine to provide that admission to the whole of the 433 beds which are at present maintained by the King's Fund shall be free to the deserving poor without tickets or payment of any kind is a fact well calculated to extend their influence and popularity.

We could wish that more members of the public would take an opportunity to visit some of the older hospitals like Guy's, the London, and St. George's that they may realise the vast improvements brought about in the older buildings by reconstruction and renewals—a change which London owes in no small measure to the institution of the King's Fund. The momentous changes which we already owe to the initiative of the King and the Prince of Wales through this Fund afford the best guarantee that the voluntary hospital system in this country will continue its successful career and be accompanied by such developments as scientific discovery and the needs of the population may show to be desirable in the public interest. All has so far been accomplished without interfering with the individual efforts of the managers of each institution with whom it is desirable that the responsibility of raising the bulk of their revenue from voluntary sources should continue to rest. It is a great fact that in five years, during a period when other and pressing claims have absorbed so much of the money of the benevolent, the King's Fund should have steadily grown to its present amount. But above and beyond this we feel that the recognition of the privilege and practice of personal service by the King and the Prince of Wales especially has exercised an influence for good upon all classes of the community, which is calculated to benefit the nation to an extent which can hardly be exaggerated. Character, after all, is the thing which matters. A nation is but an aggregation of individuals, and the Royal examples in this matter of personal service must raise the standard of the national character. The pleasure and pride with which every Englishman now regards the action of King Edward and the Prince of Wales in initiating and personally working so hard to secure the success of the King's Hospital Fund will, we hope, prove as helpful in well doing as they are legitimate and sincere.

